# Designing a robotic smart home for everyone, especially the elderly and people with disabilities

**DOI:** 10.20407/fmj.2018-009

**Published:** 2019-02-06

**Authors:** Shigeo Tanabe, Eiichi Saitoh, Soichiro Koyama, Kei Kiyono, Tsuyoshi Tatemoto, Nobuhiro Kumazawa, Hitoshi Kagaya, Yohei Otaka, Masahiko Mukaino, Akira Tsuzuki, Hirofumi Ota, Satoshi Hirano, Yoshikiyo Kanada

**Affiliations:** 1 Faculty of Rehabilitation, School of Health Sciences, Fujita Health University, Toyoake, Aichi, Japan; 2 Department of Rehabilitation Medicine I, School of Medicine, Fujita Health University, Toyoake, Aichi, Japan

**Keywords:** Smart house, Robot, Elderly people

## Abstract

We initiated the Robotic Smart Home (RSH) project to develop a comfortable, safe home environment for all people, including the elderly and individuals with disabilities. An important consideration when introducing robots into a home environment is the confined living space, the so-called space problem. The RSH project plans to simultaneously develop robots and an architectural design for living spaces to create an optimal home environment that will help elderly people live independently at home for longer periods. The RSH accommodates the following three robotics and assistive systems: mobility and transfer assist system, operational assist system, and information assist system. The mobility and transfer assist system includes three types of devices (lifting type, lateral-transfer type, and suspension type), which can be available to users as appropriate according to the severity of their disability. The operational assist system combines a hand robot with an environmental control system for the convenience of users. An information assist system connects the RSH with remote locations for communication. Inside the RSH, a home automation and monitoring system connected to the Internet of Things provides residents with comfort and security. As part of this project, two RSH centers have been established for effective facility adoption.

## Introduction

Japan’s population is aging rapidly,^1^ and the nation is facing an era that will be characterized by “massive death and massive disability.” According to a survey by Japan’s Ministry of Health, Labour and Welfare on medical benefit expenditure, the morbidity prevalence rate rises steadily with age.^2^ Since the start of long-term care insurance in 2000, the number of Japanese requiring long-term care or support increased approximately threefold over 14 years: from 2,181,621 to 5,859,067.^3^

Curious to say, despite the decline in Japan’s overall population, the country’s number of households is increasing.^4,5^ A major contributor to that increase is the decreasing number of members in a household; it is also the result of the rising number of elderly households, which currently account for approximately 25% of the total household number.^1^ Half of elderly households consist of a single person; the other half are couples’ households. In light of this situation, it is clear that many elderly Japanese people requiring care and support live alone or with an elderly partner. It is evident that if elderly people wish to continue living at home, they will require assistive technology in the home environment. Accordingly, in 2016, we initiated the Robotic Smart Home (RSH) project to develop a comfortable, safe home environment for everyone—especially elderly people and those with disabilities.

## Simultaneous Development of Robots and the Home Environment

We expect that the RSH project will assist the activity of residents by means of robotic devices; they will operate in conjunction with home automation and monitoring systems made possible by Internet of Things (IoT) connections among devices. An IoT-enhanced home automation system is regarded as a promising area of technology to make daily living more convenient and pleasant.^6^ In recent years, so-called smart homes or smart houses have gained popularity. However, with the RSH project, it is necessary to develop smart homes designed specifically for elderly people and individuals with disabilities by optimizing various functions related to the monitoring system to be implemented in addition to other factors, such as the user interface. As well as home automation technology, assisting the activities and daily living of elderly people requires robotic devices that can provide physical assistance. Mobility and transfer assist system, and operational assist system (e.g., robotic hand functions) are essential in this regard.

When introducing robots into home environments, an important consideration is the small size of the living area, the so-called space problem. The average size of household living space in Europe and the United States is about 80 m^2^; whereas in Japan and other Asian countries, it is about half that.^7^

The most problematic space limitation in a home is the restroom because it is usually designed to be occupied by one person at a time. According to the standard established by Japan’s urban development corporations, a restroom in an apartment complex typically has an area of only 1 m^2^.^8^ If a person needs assistance when transferring to the toilet, both that person and a caregiver must share the same confined space. Introducing a transfer-assist robot into a constricted area already occupied by two people is considered impossible. Therefore, to accommodate robots in confined living spaces along with residents, miniaturizing the robots is crucial. Given the current size of miniature robots, it will be necessary to rearrange the layout of living spaces for both people and robots to coexist. For example, changes need to be considered regarding the aspect ratio of floor plans and installation point of the toilet in restrooms.

## Prime Development Devices for the RSH

To provide comfort, safety, and security, the following three robotic and assistive systems are necessary: a mobility and transfer assist system; an operational assist system; and an information assist system.

### Mobility and transfer assist system

Mobility and transfer are frequent activities of daily living (ADL); thus, disorders affecting these activities have a great impact on people’s living. In all ADL for the elderly and people with neurological or orthopedic disorders, the degree of difficulty with mobility and transfer activities is second only to bathing-related activities.^9^ We are currently developing three types of mobility and transfer assist systems (lifting type, lateral-transfer type, and suspension type) that can be used according to the severity of disability.

### Lifting-type system for people with severe disability

A person with severe disability is defined as someone with limited ability to maintain a seated posture and inability to stand by themselves from a sitting position. Many cases of this type of disability have a transfer item score of 1–2 with the Functional Independence Measure (FIM). The score of 2 signifies “maximal assistance (subject 25% +)” and 1 is “total assistance (subject 0% +).” People in this category are suitable for using a lifting-type system, and this has already been proposed for medical and nursing care facilities.^10–12^ With one common type of lifting-type system, care receivers are lifted from the front of the body and their weight is supported by means of actuators. After being lifted, they can be transferred to the desired location, such as a restroom or bathroom. The lifting-type system is useful for easy transport of people with severe disability. However, the size of such systems is relatively large for use in an average-sized housing space, particularly in Asia. Thus, it is necessary to miniaturize this type of system to be applicable in a home environment.

### Lateral-transfer type system for people with moderate disability

A person with moderate disability is defined as someone who uses a wheelchair with or without assistance on a daily basis. Many cases of this type of disability fall under the category of an FIM transfer item score of 3–4. The score of 4 signifies “minimal assistance (subject 75% +)” and 3 is “moderate assistance (subject 50% +).” A lateral-transfer type system (an entirely new type of mobility and transfer assist system) is suitable for people in this category. This new system embodies an improvement in the transfer procedure: from the normal “getting-up transfer” to the novel lateral transfer. The getting-up transfer involves lifting the care recipient’s buttocks during the transfer from one surface to another (e.g., from a seat to another surface). By contrast, lateral transfer involves moving the buttocks laterally without lifting.

A wheelchair is an important device for mobility outside the home; however, it may not be the best mobility device for use within the home. For example, transferring into and out of a bed or a wheelchair or onto and off a toilet is a frequent demand in ADL. However, for wheelchair users, the degree of difficulty with such transfer movement tends to rank very high compared with other movements (e.g., reaching movements). In addition, the transfer motion is a major contributor to upper limb pain and injuries among wheelchair users.^13–15^ According to previous studies, the difficulty in the transfer motion is increased by the following factors: difference in height between the surfaces; width of the gap; and the presence of obstacles between the surface of a wheelchair and the target plane to which the person transfers (e.g., wheelchair armrests).^16^

**Figure 1:**
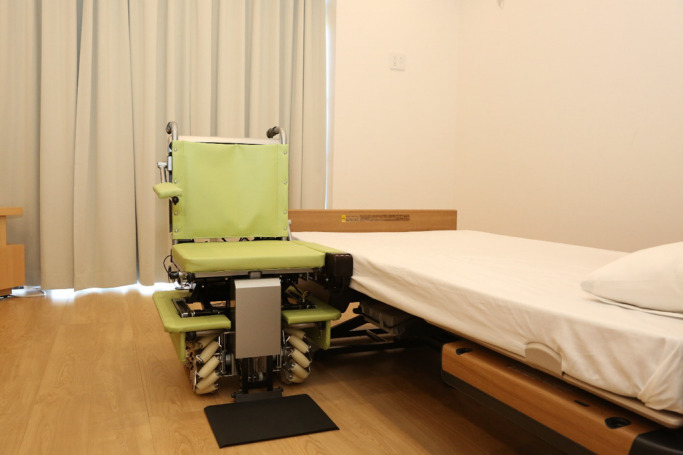
Example of a lateral-transfer type system The lateral-transfer type system has an innovative approach to performing easy transfers: electrically adjustable armrests and seat, easy rotation in any direction, ability to move easily to any location.

To address these problems, we are developing a lateral-transfer system that simplifies the transfer motion to one of lateral transfer (Figure 1). This system has various distinctive features and innovations. The seat height can be electrically adjusted to the same level as the transfer surface. The armrest automatically follows the seat’s movement and fills the gap between the seat and the transfer surface. The wheeled platform has unconstrained mobility and can move in any direction; the body of the vehicle can move very close to the transfer surface, even in confined spaces. This system reduces the user’s need to raise their center of gravity and allows them to transfer to another surface by laterally moving their center of gravity.

### Suspension-type system for people with mild disability

**Figure 2:**
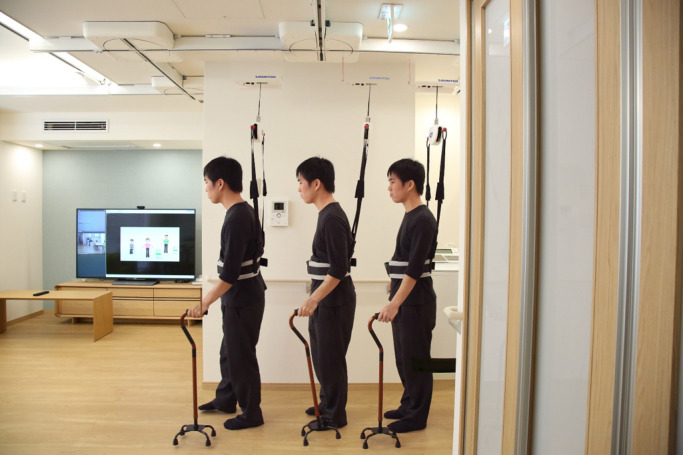
Example of a suspension-type system (walking support robot, Moritoh Co., Ltd.) This system is used to prevent falls while walking. The electric turntable system on the ceiling guides users in the desired direction by automatically moving the turntables.

A person with mild disability is defined as someone who can barely walk using walking aids and has a high risk of falling. Many cases of this type of disability have an FIM transfer item score of 5–6. The score of 6 signifies “modified independence” and 5 means “supervision.” People in this category are candidates for the suspension-type system (Figure 2). This type of system commonly consists of rails and electric or manual turntables, a lifting unit, and a harness. In general, the suspension-type system is used by health-care workers as a functional transfer assistance device to reduce the risk of injury when lifting, transferring, and repositioning patients with severe disability.^17,18^ However, our concept with the suspension-type system is different: the system is designed to be used by care receivers, and it prevents them from falling while walking without the support of their body weight (often called safety suspension). This system helps users to walk actively in ADL without fear of falling.^19–21^ Thus, our system targets people with mild disability who are at increased risk of falling. Although it is not the system’s principal use, it is possible to adjust the length of the harness electrically to change the degree of body weight support toward reducing the difficulty of independent walking. Moreover, the electric turntable system guides users in the desired direction by automatically moving the turntables when they set the destination in advance using a tablet computer or voice recognition system (e.g., from bedroom to restroom). When the patient walks to the position under the turntable, the turntable automatically rotates to the predetermined direction.

### Operational assist system

**Figure 3:**
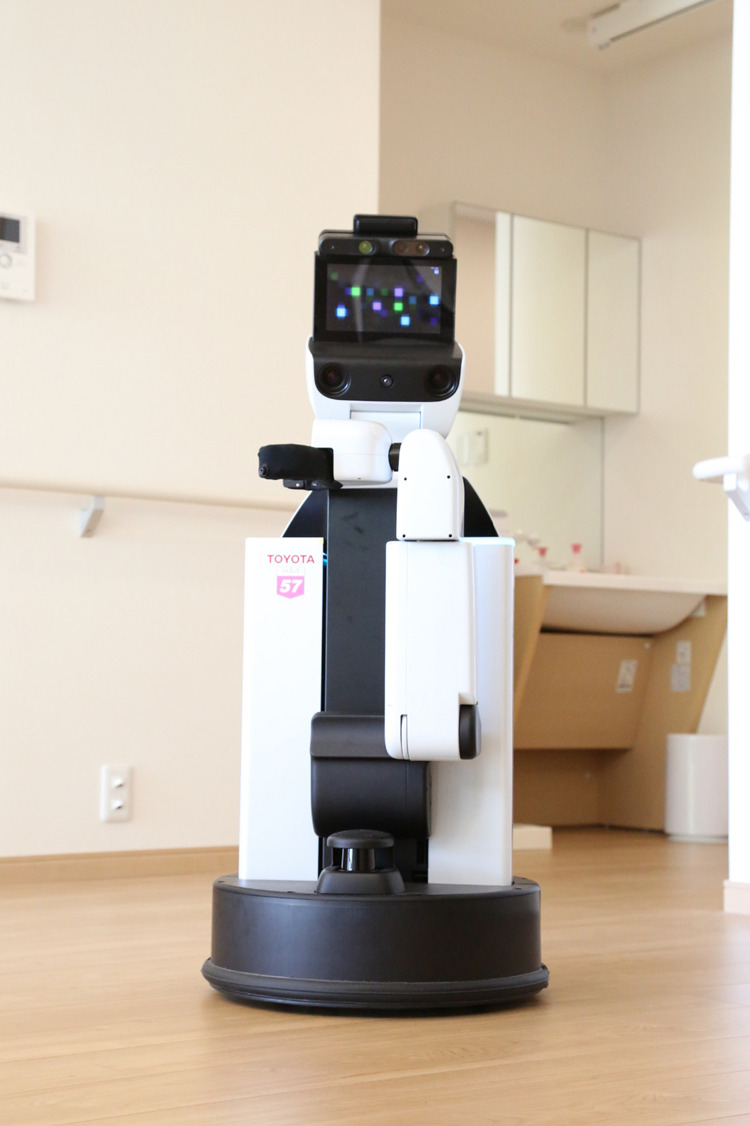
Example of an operational assist system (human support robot, Toyota Motor Corp.) This system works better with a combination of a robotic hand and an environmental control system. The robotic hand is used for non-typical tasks, such as picking up and carrying a bag or household goods; the environmental control system is used for typical tasks that involve IoT connections, such as switching on lights.

The second essential system is the operational assist system. This system works better with a combination of a hand robot and an environmental control system (Figure 3). With confined spaces, conventional housing cannot accommodate many robots along with residents; thus, the hand robot has to assume many of the key roles of ADL except for mobility and transfer assist systems; e.g., it communicates with residents and serves as a concierge or butler by operating various smart appliances in accordance with the user’s commands. The environmental control system is used for typical tasks using IoT connections (e.g., opening the curtains, turning on the TV, switching on lights, and controlling air-conditioning). The robotic hand is used for non-typical tasks, such as picking up and carrying a bag or household goods. From the perspective of rehabilitation medicine, essential manipulation tasks required for the elderly and people with disabilities are tasks related to ADL, especially self-care, e.g., dressing, eating, and toileting.^22–24^ These are important tasks, but they are not easy to achieve because the robotic hand has to move close to or touch the user.

### Information assist system

**Figure 4:**
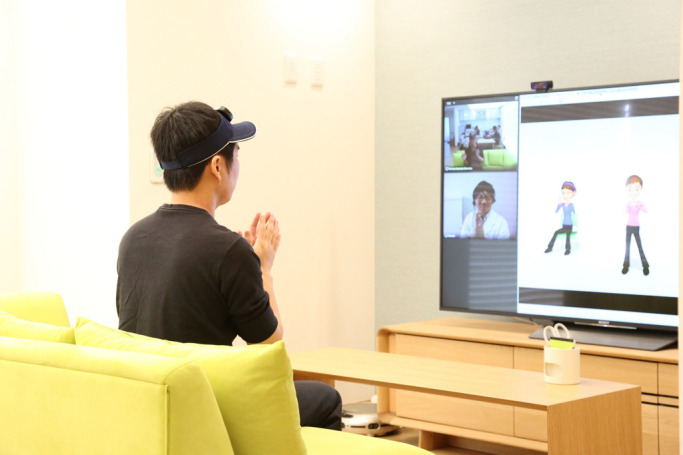
Example of an information assist system (Commu-TV, Brother Industries, Ltd.) This system may promote health in daily living. The television-type communication device allows users to communicate with staff at a remote medical institution, and it can provide effective, enjoyable tele-exercise programs enhanced with computer graphics.

The final essential system is the information assist system. Specifically, that system connects the RSH with remote sites for communication. An IoT-connected home automation and monitoring system within the RSH provides residents with comfort and security. One innovative area with the information assist system is that it may promote good health in daily living. For example, wearable monitoring systems measuring a person’s real-time activity and heart rate could be useful for lifestyle guidance with respect to that person’s physical functions and amount of daily activity. A television-type communication device allows users to communicate with staff at a remote medical institution. This communication system provides effective, enjoyable tele-exercise programs enhanced with computer graphics (Figure 4). Real-time monitoring of physiological conditions (heart rate and blood oxygen saturation) collected by wearable sensors can be used for exercise-tolerance tests and appropriately adjusting exercise intensity to ensure safety for people with respiratory and cardiovascular disease. Conceptually, a health check toilet can be used to prevent lifestyle-related diseases based on urine measurements. For example, a salinity measurement could provide valuable information for preventive low-salt dietary instructions in pre-hypertensive patients.

## Practical and Functional Facility of the RSH

Various types of projects have been conducted toward developing smart homes for the elderly.^25,26^ However, insufficient facility utilization has frequently been observed. Effective facility adoption involves two aspects: authentic assessment and interactive development. To accommodate each aspect, we have opened two centers: an experimental center for RSH (RSH-EC) and a development center for RSH (RSH-DC).

**Figure 5:**
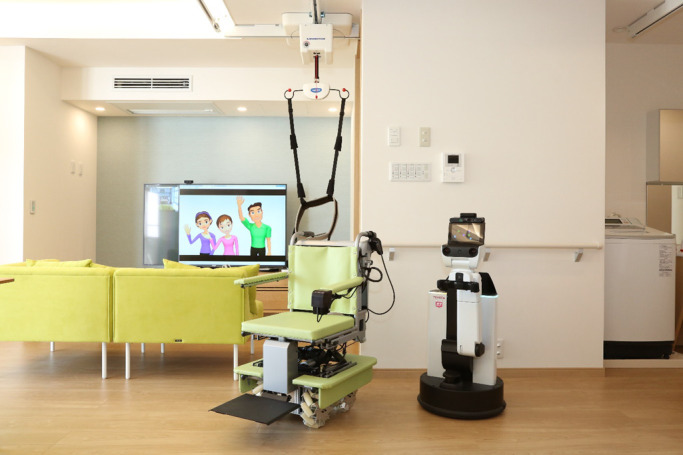
Living-dining room in the RSH facility constructed in an apartment complex The experimental RSH center is situated within the local community where potential users reside. As a first step, we have used a slightly larger room size (about 75 m^2^) to investigate the impact of layout rearrangement.

With authentic assessment, it is important is to establish an experimental facility within the local community where potential users reside. Many test or demonstration facilities in the past were set up in places that potential users could not easily access from their homes. The maximum walking distance that elderly people can usually tolerate is 30 minutes.^27^ To ensure convenient access for local elderly residents, the RSH-EC was established within an apartment complex located close to our university (Figure 5). Many elderly people already live there: among approximately 2,000 households with 4,000 residents, there are 1,300 people aged over 65 years. In addition, a branch of the university’s center for comprehensive community care is also located in that apartment complex. The staff include physical therapists, occupational therapists, and nurses; they have been providing health consultation and lectures to residents and have established a close relationship with them. Based on authentic, real-world assessment, our medical staff collects and analyzes information about elderly people’s needs. The staff subsequently collaborates closely with industry engineers to define the rationality and mechanisms for companies to develop and improve assistive devices.

The other factor that affects facility underutilization is an interactive development system that effectively utilizes facilities with different functions. In addition to the RSH-EC in the apartment complex, the RSH-DC has been constructed within our campus. The RSH-DC focuses on promoting emerging technologies in collaboration with many companies toward developing future assistive technologies for implementation in the RSH-EC. No single system can cover all needs of the elderly. By developing various systems for different purposes at the same place, the systems can interact with one another. As a result, useful functions arise, and unnecessary functions are eliminated. Eventually, the systems are combined as an integrated package for the RSH.

With both RSH centers, a symbiotic platform has formed to promote a collaborative partnership among our university, member companies of the RSH project, and many other companies. The whole community is now accelerating the development of various devices. These RSH centers are expected to generate synergistic effects. While authentic assessment is being conducted at the RSH-EC in the apartment complex, engineers continue improving devices at the RSH-DC within the university. In addition, newly developed devices that demonstrate the effectiveness at the RSH-DC can be introduced at the RSH-EC for assessment by potential users.

## Conclusions

Rehabilitation robots can be described as activity assist robots (AARs): the aim of rehabilitation is to promote activity. AARs are divided into four categories: independent assist,^28–30^ exercise assist,^31,32^ care assist,^10^ and cognition/emotion assist.^33^ The RSH addresses independent assist, care assist, and cognition/emotion assist. With the RSH project, an innovative collaboration model among the university, companies, and public institutions has been proposed. The participants include physicians, physical therapists, occupational therapists, robotics engineers, information technology engineers, architectural designers, administrative officials, and potential users. This novel model has the potential to increase adoption and achieve success. In a longevity society with fewer children, RSH could bring great happiness to everyone, including the elderly and people with disabilities.
